# Atmospheric Measurements by Ultra-Light SpEctrometer (AMULSE) Dedicated to Vertical Profile in Situ Measurements of Carbon Dioxide (CO_2_) Under Weather Balloons: Instrumental Development and Field Application

**DOI:** 10.3390/s16101609

**Published:** 2016-09-29

**Authors:** Lilian Joly, Rabih Maamary, Thomas Decarpenterie, Julien Cousin, Nicolas Dumelié, Nicolas Chauvin, Dominique Legain, Diane Tzanos, Georges Durry

**Affiliations:** 1 Groupe de Spectrométrie Moléculaire et Atmosphérique GSMA, Université de Reims—Champagne Ardenne, UMR CNRS 7331, Moulin de la Housse, BP 1039, 51687 Cedex 2 Reims, France; thomas.decarpenterie@univ-reims.fr (T.D.); julien.cousin@univ-reims.fr (J.C.); nicolas.dumelie@univ-reims.fr (N.D.); nicolas.chauvin@univ-reims.fr (N.C.); georges.durry@univ-reims.fr (G.D.); 2 Groupe d’étude de l’Atmosphère Météorologique, Centre National de Recherches Météorologiques, UMR 3589, Météo-France/CNRS, 42 Avenue G. Coriolis, 31057 Cedex 1 Toulouse, France; dominique.legain@meteo.fr (D.L.); diane.tzanos@meteo.fr (D.T.)

**Keywords:** greenhouse gases, atmosphere, lightweight spectrometer, meteorological balloons, flights, vertical profiles, in situ

## Abstract

The concentration of greenhouse gases in the atmosphere plays an important role in the radiative effects in the Earth’s climate system. Therefore, it is crucial to increase the number of atmospheric observations in order to quantify the natural sinks and emission sources. We report in this paper the development of a new compact lightweight spectrometer (1.8 kg) called AMULSE based on near infrared laser technology at 2.04 µm coupled to a 6-m open-path multipass cell. The measurements were made using the Wavelength Modulation Spectroscopy (WMS) technique and the spectrometer is hence dedicated to in situ measuring the vertical profiles of the CO_2_ at high precision levels (σ_Allan_ = 0.96 ppm in 1 s integration time (1σ)) and with high temporal/spatial resolution (1 Hz/5 m) using meteorological balloons. The instrument is compact, robust, cost-effective, fully autonomous, has low-power consumption, a non-intrusive probe and is plug & play. It was first calibrated and validated in the laboratory and then used for 17 successful flights up to 10 km altitude in the region Champagne—Ardenne, France in 2014. A rate of 100% of instrument recovery was validated due to the pre-localization prediction of the Météo—France based on the flight simulation software.

## 1. Introduction

Water vapor (H_2_O), methane (CH_4_) and carbon dioxide (CO_2_) are major greenhouse gases (GHGs) with a strong impact on the climate. CO_2_ is one of the main boosters of GHGs since the beginning of the industrialization era due to anthropogenic activities, and it is considered as the main cause of global warming [1]. Anthropogenic activities such as fossil fuels, ruminant emissions, and biomass burning constitute the major sources of carbon dioxide and methane. The increase of H_2_O concentration [2] in the stratosphere could cause a cooling of this atmospheric region, impacting the recovery of the ozone layer [3]. Therefore, having information and data about the vertical distribution of H_2_O, CO_2_ and CH_4_ is very useful to improve our knowledge of the future of our climate.

It has been widely recognized, with virtually unanimous agreement from scientists, that the Earth’s atmosphere is growing warmer due to greenhouse gas emissions generated by human activity. The aim of the Paris Conference (http://www.cop21.gouv.fr/) held in December 2015 was to come up with a response to this problem. According to the evidence of climate change and that the radiative effects in Earth’s climate system is related to the atmospheric composition in the upper troposphere and the lower stratosphere [4,5], one should improve the knowledge/estimation of the regional anthropogenic greenhouse natural sinks and emission sources for a better quantification. This goal becomes possible: (1) by enhancing the atmospheric chemistry-transport models that are used to connect sources and sinks to atmospheric concentration; and (2) by increasing the atmospheric observations (satellite, aircrafts, balloons, …) in addition to ground measurements such as the Integrated Carbon Observation System (ICOS, https://www.icos-ri.eu/). Moreover, an increase of in situ measurements which are complementary to satellite-measured data (for accurate and precise concentration scaling) is highly required.

A vertical concentration distribution up to a few tenths of a kilometer can nowadays be obtained by techniques such as remote sensing (lidars, radiometers) or through in situ measurements such as aircraft, drones, open stratospheric balloons (BSO) and meteorological balloons. Each technique has specific advantages and disadvantages. Balloon systems, for example, can reach higher altitudes at lower speed than aircraft, which is essential for higher spatial-resolution measurements, while their random trajectories are not well defined, in contrast to aircraft. On the other hand, the lighter the weight of the instrument, the easier the logistic preparation and authorization for flights under smaller balloons [6]. Moreover, the average price of a meteorological balloon flight is much cheaper than that of BSO balloons and aircraft flight. Therefore, small balloons are considered as a good option to help understand greenhouse gas emission processes.

Since an increase in the atmospheric greenhouse gas distribution is crucial, we report in this paper the development of a lightweight instrument that complies with the requirements for a flight from weather balloons. This instrument is dedicated to in situ measurements of carbon dioxide up to 10 km altitude in order to study and understand the Atmospheric Boundary Layer (ABL) levels. This instrument could further be useful to study the coupling process between the upper troposphere and the lower stratosphere. Hence, Schmidt and Khedim have reported in situ CO_2_ data obtained with a balloon-borne cryogenic sampler [7]. These measurements have revealed a deficiency of a few tenths of ppm between the upper troposphere and the lower stratosphere that could be related to the transport and mixing processes coupling those two altitudes. The main science objective of this instrument is to contribute to the validation of the space (satellite) mission devoted to the monitoring of carbon dioxide in the Earth atmosphere. To achieve higher precision measurements and a spatial resolution of a few meters in the vertical in situ concentrations profiles, one should combine balloons and diode-laser spectroscopy techniques. To this end, we have developed optical sensors based on near-infrared absorption spectroscopy [8] which provide a compact, cost effective, fully autonomous, low-power and non-intrusive probe to measure carbon dioxide in the atmosphere using an open-path multipass cell.

## 2. Atmospheric Spectroscopy

Diode Laser spectroscopy provides interesting advantages related to its high selectivity and sensitivity in the detection of gases. Semiconductor diode lasers emitting in the near infrared (NIR) have played an important role because of their continuous mode and tunablity as well as their relative low amplitude noise. The main reason is that most atmospheric pollutant molecules feature suitable absorption lines in the near infrared spectral region.

### 2.1. Direct Absorption Spectroscopy (DAS) Technique

The simplest realization of laser-based absorption techniques, and therefore well adapted to in situ measurements, is Direct Absorption Spectroscopy. The beam of a tunable laser passes through a gas sample and then is measured with a detector. If the frequency of the emitted light is close to a molecular transition of the gas sample, the light is then absorbed and the transmitted intensity decreases. The concentration of the absorbing species in the gas mixture is then calculated according to Lambert-Beer’s law [9].

### 2.2. Wavelength Modulation Spectroscopy (WMS) Technique

**Figure 1:**
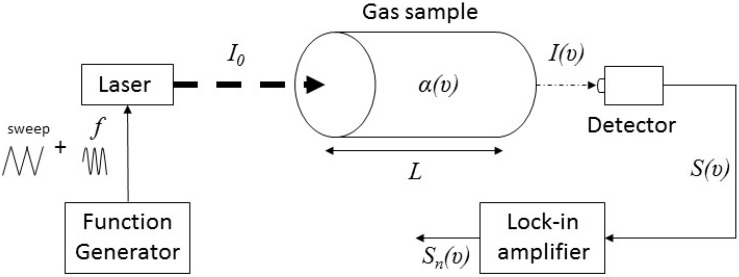
WMS basic principle. The incident laser light (*i*_0_) that passes through the gas sample having an absorption coefficient (*α*(*υ*)) on a defined path-length (*L*) is modulated at a specified frequency (*f*). The transmitted light is then attenuated by the absorption (*I*(*υ*)) and measured by the detector (*S*(*υ*)). This latter is demodulated at an integral multiple (*n*) of the modulation frequency in order to obtain a demodulated signal (*S_n_*(*υ*)). The function generator is used to sweep the laser frequency in order to obtain an absorption spectrum of the gas.

By shifting detection to higher frequencies using modulation techniques [10] the 1/*f*-noise can be reduced. WMS is the most common of these techniques. It is advantageous for applications with small absorbance at atmospheric pressures. The frequency of the laser is modulated at high *f* values (in the kHz range) leading to a modulation of the transmitted intensity near the transition. The detected signal is then demodulated at a multiple integer *nf* of the modulation frequency using a lock-in amplifier. This technique increases the sensitivity [11,12,13,14] compared to direct absorption spectroscopy described above. Figure 1 shows the basic principle of this WMS technique.

The principle of our developed spectrometer is based on the WMS technique which allows us to attain high-sensitivity detection. Besides that, working at atmospheric pressures is necessary in such light-weight instruments. Furthermore, second harmonic detection produces a zero baseline signal, thus eliminating the necessity of measuring small differences between two large intensities, *I*_0_ and *I*(*υ*) (Figure 1), as in the case of direct absorption spectroscopy [15]. Therefore, the WMS technique allows us to overcome baseline artifacts and background measurements.

All of the *nf* components of the WMS signal are proportional to laser intensity. At the absorption line center, the WMS-1*f* term is dominated by the laser intensity contribution [16]. Generally, the second harmonic (2*f*) is used because it is strongly dependent on spectral parameters and gas properties and can therefore be compared with spectral simulations to infer gas properties. Simultaneous wavelength and intensity modulation for the emitted laser light enables WMS-1*f* normalization of the WMS-2*f* signal to avoid the need to scan on and off the transition for the zero absorption baseline used in direct absorption spectroscopy technique which leads to non-absorption losses in laser power due to scattering, window fouling or laser power drift [11,12]. This normalization allows quantitative measurements without determining a zero-absorption baseline which makes WMS an attractive technique for absorption measurements in harsh environments (high pressure, high temperature, high-opacity, …) [11]. Red and Labrie [17] have demonstrated that the magnitude of the WMS-2*f* signal is related to the ratio of the modulation depth of the laser to the half-width at half-maximum (HWHM) of the selected spectral line. When this ratio is equal to 2.2 at a fixed pressure and temperature, the WMS-2*f* signal reaches its maximum.

## 3. Instrument Development

In this section we present the Atmospheric Measurements by Ultra-Light SpEctrometer (AMULSE) which is an open-path tunable diode laser-based sensor. The laser emits in a continuous mode in the near-infrared spectral region of 2.04 µm. This spectrometer is dedicated to measurements of carbon dioxide mixing ratio in the atmosphere by applying the WMS technique described above.

### 3.1. Line Selection

**Figure 2:**
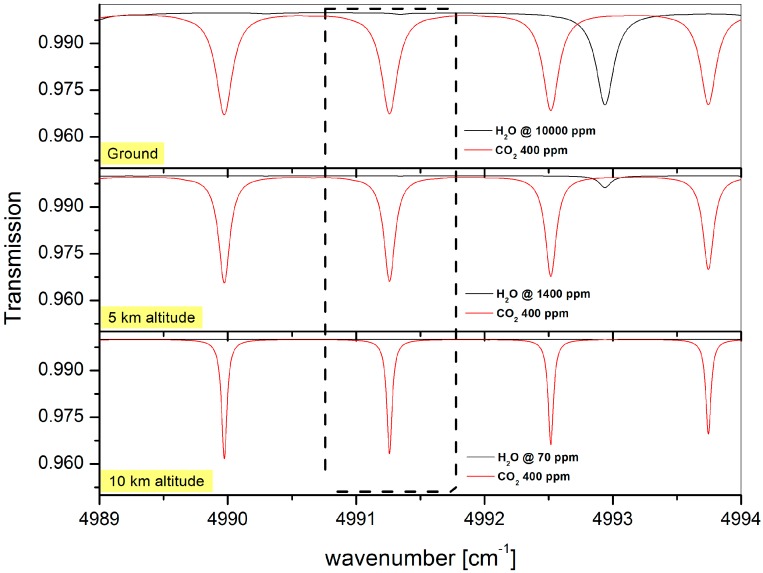
Simulated absorption spectra ranging from 4989 cm^−1^ to 4994 cm^−1^ for an optical path-length of 6 m at ground level (1050 mbar, 20 °C), 5 km altitude (660 mbar, 0 °C) and 10 km altitude (275 mbar, −45 °C) for the carbon dioxide absorbing at 400 ppm and the water vapor absorbing at natural concentrations [19]. The selected carbon dioxide transition (in the dashed-line box) is sufficiently isolated for atmospheric sensing (at 4991.26 cm^−1^) and has no interferences with other CO_2_ transitions nor H_2_O transitions.

The targeted CO_2_ absorption line (Figure 2) is well isolated (free from interferences with other molecules and close absorption lines of carbon dioxide) at 4991.26 cm^−1^. This ro-vibrational transition belongs to the R-branch of the 20012-00001 (ν_1_ + 2ν_2_ + ν_3_) combination band [18].

### 3.2. Optical Setup

The AMULSE laser is a GaSb-based DFB semiconductor diode in a butterfly package (nanoplus GmbH, Gerbrunn, Germany). The optical power at the output of the single mode fiber is approximately 1 mW at a current of 95 mA. A fiber-based collimator in a pigtail style (LPC-01-2004-7/125-S-1-2.61GR-40-3A-3-1, Oz Optics, Carp, ON K0A 1L0, Canada) with a GRIN lens (2.61-mm focal length) is connected to the output of the laser optical fiber in order to collimate the light (1-mm beam diameter). This light is directed to a home-made open-path multipass cell configured as Herriott type. The focal length of the used mirrors (2-inche diameter) is equal to 150 mm. Small (2-Watt) heaters are attached on the edge of each mirror in order to prevent condensation during balloon descent due to the low temperature of the high troposphere. One hole on each mirror is drilled to attach the optical-fiber collimator on the input mirror side, and the 1-mm diameter InGaAs photodetector (J23-18D, Judson, Montgomeryville, PA, USA) on the output mirror. The effective optical path length is 6 m for a base length of 19.5 cm. The tunable diode laser is set to emit near the selected CO_2_ absorption line by controlling its temperature and the injected DC current (resulting in a 1.95 cm^−1^ spectral interval). A sinusoidal modulation current at 4.5 kHz is introduced superimposed to 5 Hz saw tooth current ramp (very slow compared to the modulation frequency) which sweeps the laser wavelength across the absorption feature.

### 3.3. Embedded System

**Figure 3:**
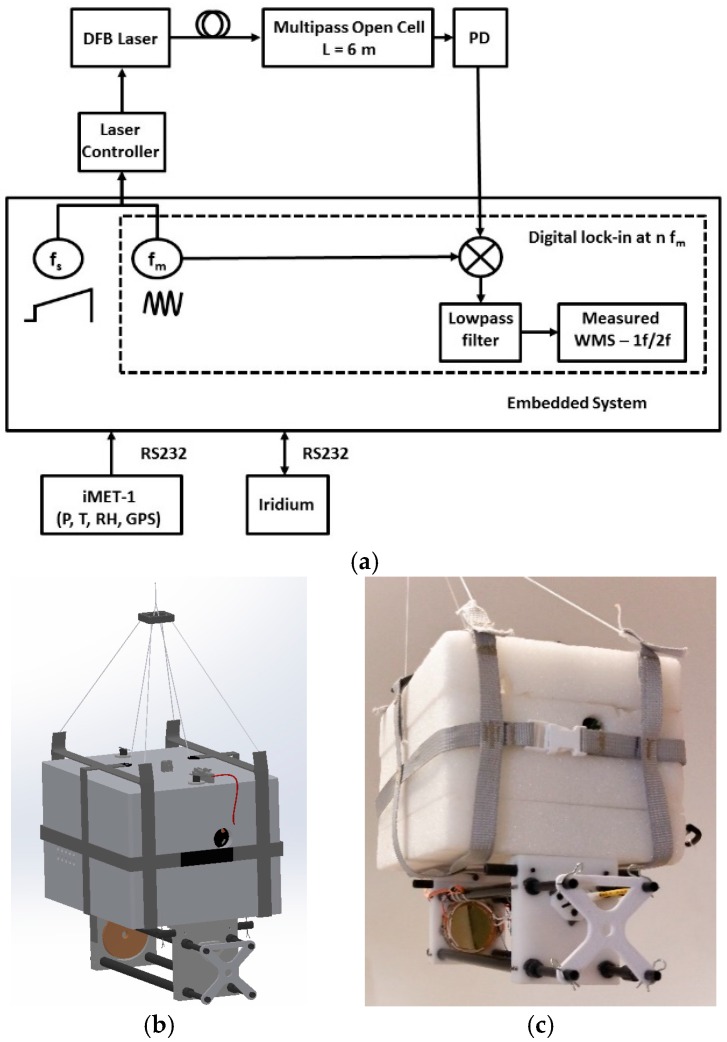
(**a**) Full schematic diagram of the developed AMULSE in the embedded system configuration; (**b**) 3D drawing of the final version of the AMULSE. The dimensions of this instrument are 290 × 175 × 227 mm; (**c**) Photography of the AMULSE taken after the measurement campaign.

Figure 3a shows the complete embedded system scheme of the AMULSE. The main electronic board of the sensor is a single National Instruments (NI, Nanterre, France) RIO-9636 board. The system is powered from a single +8 VDC Li-ion battery (rechargeable, 7800 mAh) dispatched through regulators that are well adapted to the different parts and components. The total power consumption for the electronics, excluding the mirrors heaters, is equal to 8 W. The digital lock-in/data acquisition system is integrated into the FPGA unit on the NI sbRIO-9636. It generates the laser modulation waveform and applies it to the home-made current/temperature laser driver. The modulated signal from the photodetector is digitized into 8192 sample points using a 16-bit digitizer from the NI single board. After processing by the digital lock-in FPGA system, WMS-2*f*, WMS-1*f* and direct signals are stored onboard into binary files in a SDHC memory card.

The atmospheric pressure and temperature are monitored by iMET-1-RS radiosondes (International Met Systems, Grand Rapids, MI, USA) equipped with RS232 interfaces. Temperature accuracy given by the head thermistor is equal to 0.2 °C while the pressure accuracy given by the piezo-resistive sensor is equal to 0.5 mbar. The location of the sensor in flight is obtained from the iMET-1 onboard GPS. Sensor location, monitoring parameters, WMS-2*f* and WMS-1*f* signal amplitudes and atmospheric pressure and temperature are transmitted to the ground through a 2-way satellite communication system (Iridium).

The strap (ON/OFF button) plug-and-play spectrometer weighs 1.8 kg in flight-ready condition, including cabling, thermal insulation and power supplies to be autonomous for 8–9 h according to ground tests. The instrument is compact (290 × 175 × 227 mm) as can be shown on Figure 3b,c.

### 3.4. AMULSE CO_2_ Setup

One lock-in multiplies the detector signal by a reference sine wave (an integer of the laser modulation frequency) in order to obtain the Y component of the signal while the other multiplies the detector signal by a corresponding cosine wave to obtain the X component of the signal. The root-sum-square of the X and Y components is the total magnitude of the selected harmonic.

Assuming linear intensity modulation with a phase shift of π, the 1*f*-normalized WMS-2*f* signal simplifies to the following equation [11,12]:

(1) $$\frac{2f}{1f}=\frac{S(T)\times P\times x_{i}\times L}{i_{0}\times \pi }\int_{-\pi }^{+\pi }\Phi \left(\nu_{peak}+a\cos\left(\theta \right)\right)\times \cos2\theta d\theta$$

where *S*(*T*) is the line strength at temperature *T* of the absorption transition, *P* is the partial pressure of the target species (CO_2_ in this case), *x_i_* is the target species concentration (to be fitted), *L* is the path length of laser beam travel through the uniform absorbing medium, *i*_0_ is the amplitude of the linear term of the laser intensity modulation (to be determined beforehand experimentally in the laboratory) [16], *ν_peak_* is the peak central frequency position, *a* is the amplitude of the frequency (wavelength) modulation, θ corresponds to *ωt* or 2π*ft* and Φ is the line shape function at the absorbing frequency (in our case we used a Lorentzian profile since we are working at pressures greater than 200 mbar).

The ratio of 2*f*/1*f* signals is thus calculated for a range of temperatures at the measured pressure and at a nominal expected value of *x_i_*.

## 4. Validation and Calibration

Wavelength modulation spectroscopy signal (whether the WMS-1*f* or the WMS-2*f* signal) depends on the laser characteristics (such as emitted wavelength, applied current, laser temperature, modulation amplitude and modulation depth of 2.2 on which the signal was optimized) and on the absorbing molecule parameters (concentration, temperature, pressure). Therefore, the need for a well-controlled and automated environment is highly recommended. From one side it is interesting to test the electronic board in some extreme conditions such as very low pressures and temperatures. From the other side it is also important to monitor any influence of temperature and pressure variation on the quality of the spectra and/or the precision of the spectrometer.

### 4.1. Atmospheric Enclosure

**Figure 4:**
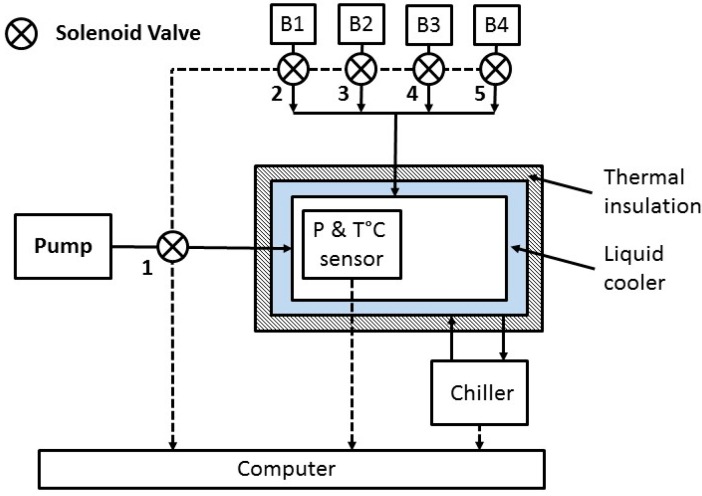
Atmospheric enclosure schematic diagram showing the thermal insulation and liquid cooler layers as well as the different elements connected and controlled by a single computer.

A detailed schematic diagram of the home-made atmospheric enclosure is presented in the following figure (Figure 4). Atmospheric chambers provide a controlled environment to study the response, stability, precision and accuracy of the laser-based instrument at a stable gas temperature, pressure and concentration. A home-made enclosure was made from stainless steel for spectroscopic applications (vacuum, extreme temperatures, non-corrosive, …). The tightness is insured by a joint fixed on the lid. This atmospheric enclosure is double-walled to be thermalized with a cryogenic fluid (glycol allowing temperature control from −25 °C to +50 °C) injected continuously by a FP50-MA Chiller (Julabo, Seelbach, Germany). Temperature stabilization is carried out by a home-made PID software system. Temperature measurements are based on four PT-100 class 1/10 temperature sensors connected to a 24-bit thermometer. A thermal insulation layer covers the whole enclosure to avoid heat-flux exchange with the outside ambient atmosphere. Five electronic valves were connected to this enclosure dedicated for a pumping system (valve number 1 on the Figure 4 below) and different calibrated gas cylinders provided by the by the “Laboratoire des Sciences du Climat et de l’Environnement LSCE” (connected separately to the valves 2 to 5 (see Figure 4)). A MKS 626A-13TDE pressure transducer having a range of 0–1000 Torr is connected to continuously control the interior gas pressure. The range of this pressure transducer is sufficient for the calibration process of the instrument. All of those elements listed above (electronic valves, pressure transducer, temperature sensors) were connected to the computer via a USB-TEMP-AI by Measurement Computing (Norton, MA, USA) in order to be controlled by our home-made-developed automated command and control software. This software is also used to turn on and control the AMULSE. This system allows variation of the pressure between 0 and 1200 mbar (with fine tuning function) for a temperature range between −25 °C and +50 °C. The standard deviation of the pressure and the temperature measurement (0.01 mbar and 0.01 °C respectively) were held at 1 Hz during 1.5 h.

### 4.2. Calibration Protocol

First, the signal (WMS-2*f*) is maximized at a modulation index of *m* = 2.2 (modulation amplitude is equal to 0.23 cm^−1^) for 660 mbar at 0 °C which corresponds to the median value of the pressure between the ground (~1000 mbar) and 10 km altitude (~200 mbar). The AMULSE is then ready to enter the calibration process inside the atmospheric chamber described above. The following procedure is based on applying three consecutive purges of the enclosure using an absorption-free gas cylinder, then on injecting different pressure levels of a well-known CO_2_ concentration. Once the temperature for each pressure level is stabilized (<0.01 °C), the WMS signal (2*f*/1*f*) is recorded during five minutes at a rate of 5 Hz. This procedure is repeated for four different CO_2_ concentrations (370.72 ± 0.02 ppm, 383.52 ± 0.01 ppm, 424.3 ± 0.01 ppm and 435.98 ± 0.01 ppm) at twenty-one evenly spaced pressure levels ranging from 200 to 1200 mbar and for eleven evenly spaced temperatures ranging from −20 °C and +30 °C.

**Figure 5:**
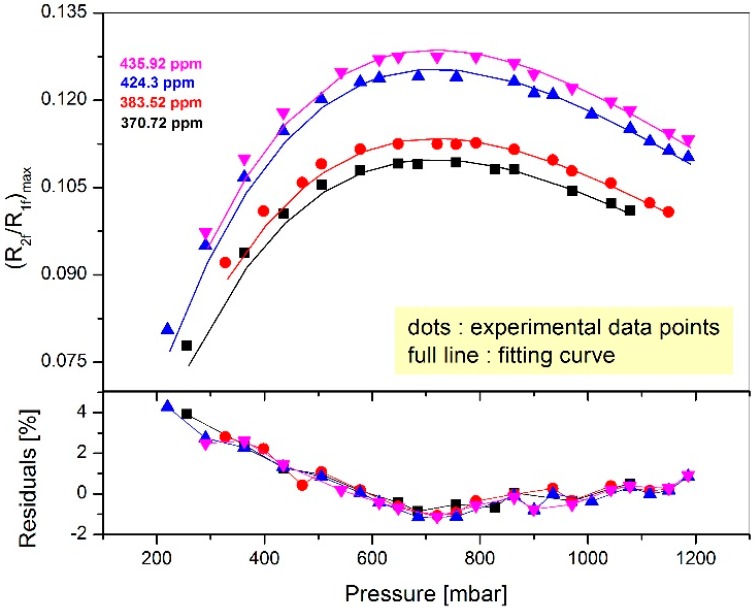
Experimental data at 15 °C, represented by points on the upper panel and their corresponding fit to the model described by Riecker [11,12] in continuous lines for the four different concentrations tested in the atmospheric enclosure. The lower panel shows the residuals of the applied fitting. The residual is about 4% at lower pressures and is reduced at 1% for the pressures around 660 mbar which correspond to our optimized modulation depth of 2.2. This fitting technique is limited by the Lorentzian profile which works better at higher pressures.

Figure 5 shows an example of the results obtained at 15 °C (experimental data are represented by points) for the four different concentrations previously listed. We determine then the maximum of each of those curves in order to obtain the corresponding modulation amplitude (*a* = 0.232 ± 0.009). Fitting the experimental data points, using the Equation (1) described by Riecker [11,12] and Reid [17] allows us to determine the *i*_0_ value (*i*_0_ = 0.049 ± 0.001). The least-square is then applied by minimizing the difference between our measured 2*f*/1*f* signal and the calculated one using the model described by the Equation (1) leading to absolute concentration determination.

### 4.3. Instrument Characterization

**Figure 6:**
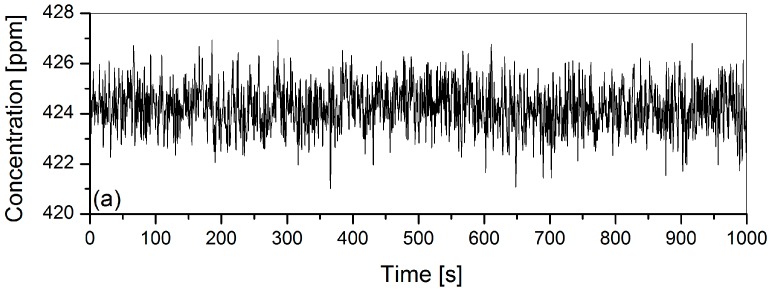

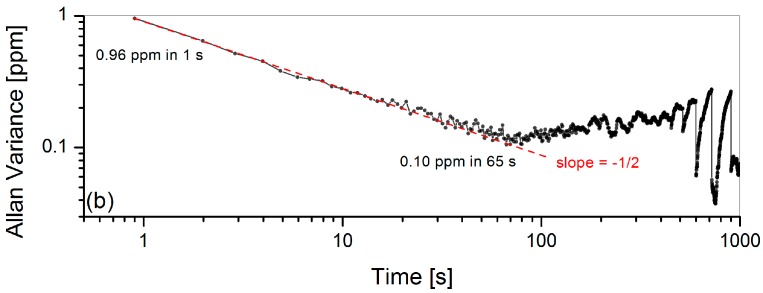
(**a**) Time series measurement; (**b**) Allan deviation plot on a logarithmic scale showing two different behaviors. First, a linear decay at the beginning (slope = −1/2) indicating linear decrease in deviation with the averaged number of the data, i.e., efficient white noise reduction and hence improvement in the measurement precision. Second, the instabilities of the instrumental system nevertheless counterbalance the noise reduction given by average and the deviation increases due to undesired drift noise.

Once we have determined *i*_0_ and *a* using the method presented by Rieker [11,12], we proceed to the instrument characterization process. First we flush the enclosure with zero-air in order to remove the memory effect, then we inject another well-determined concentration of CO_2_ from a cylinder (424.3 ± 0.02 ppm) at a fixed pressure (682.67 ± 0.01 mbar) and a fixed temperature (−0.25 ± 0.02 °C) for about 90 consecutive minutes. Time-series measurement was carried out for an Allan variance study [20,21,22]. The average concentration value determined was 424.48 ± 0.93 ppm (Figure 6a). The uncertainty of the measurement was less than 0.3%. The Allan deviation σ is plotted vs. time on a logarithmic scale (Figure 6b).

### 4.4. Validation and Intercomparison

**Figure 7:**
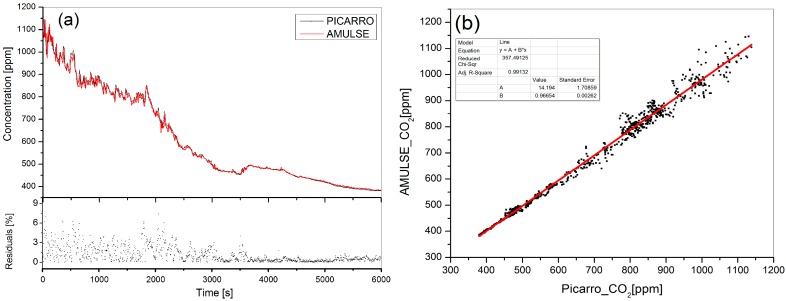
(**a**) Picarro vs. AMULSE intercomparison. On the upper panel Picarro’s data are plotted in black while AMULSE’s data are in red. The lower panel shows the residual uncertainties which are 7.39% at the higher concentrations and at the beginning of the intercomparison and about 0.01% at lower concentrations. The standard deviation of the uncertainty is equal to 1.2% for all the intercomparison; (**b**) The correlation between the AMULSE and Picarro’s measurements. A linear fit was applied with an adjusted R^2^ of 0.99132.

In situ measurements of CO_2_ were made using a Picarro CRDS (Cavity Ring-Down Spectroscopy) CO/CO_2_/CH_4_/H_2_O analyzer (model G1301, Picarro Inc., Santa Clara, CA, USA) [23]. After being calibrated, the analyzer pulled air from a wing-mounted inlet continuously at few centimeters from the AMULSE multipass cell for about 6000 s. Measurements from both instruments were averaged for 5 s. Full Picarro measurement (Figure 7a black line on the upper panel) shows a good agreement with the AMULSE measurements over all the concentration range with a minimum residual uncertainty of 0.01% at lower concentrations and 7.39% at higher concentrations. Figure 7b shows the linear correlation between the two instruments involved with an adjusted R^2^ of 0.99132.

## 5. Measurement Campaign

### 5.1. Description of the Flight Chain

**Figure 8:**
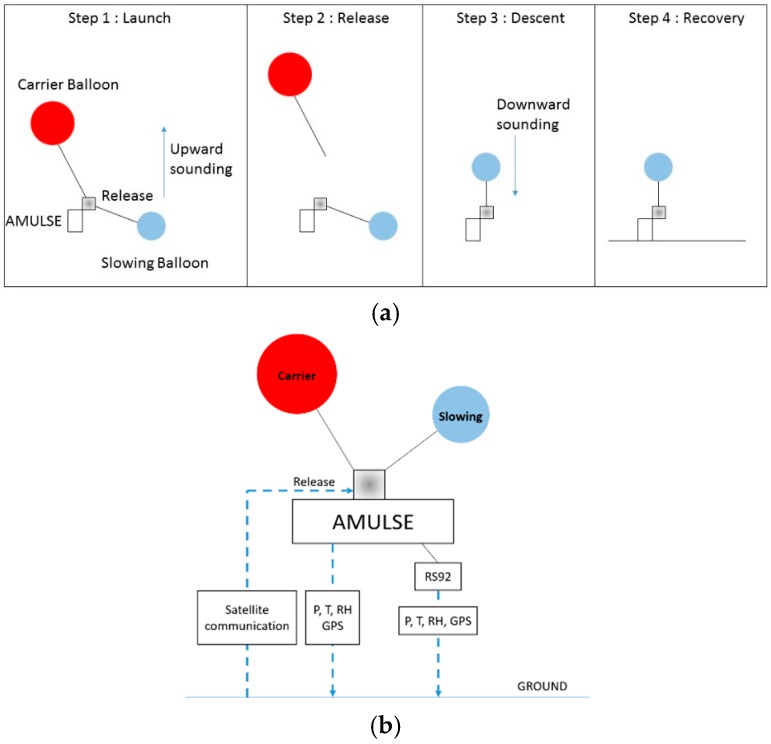
(**a**) A schematic diagram of the four steps showing the principle of the dual-balloon sounding [24]; (**b**) Transmission/Reception of the information during the flight. A Vaisala radiosonde RS92 is connected in order to send instantaneous P, T, relative humidity and GPS data to a ground mobile station to inject those data in the trajectory model and hence ensure the tracking/recovery of the instrument. Note that the trigger release system is controlled by a home-made smartphone application via satellite communication “Iridium”. The latter transmits the GPS position, pressure and temperature data every 5 min that ensure redundant retrieval before the probe lands.

**Figure 9:**
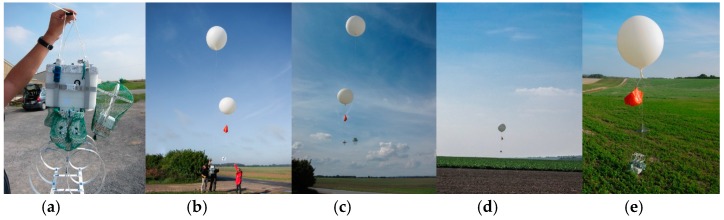
The different flight stages of AMULSE. (**a**) The instrument is securely attached to the two-balloon system and ready to fly. Stages (**b**,**c**) corresponds to the beginning of the flight while (**d**) shows the pre-positioning determined by the flight simulation software in order to recover the instrument (**e**).

Legain et al. [24] have developed a sounding system that combines both the flexibility of the free balloons and the ability to recover the instrumentation such as with tethered balloons. This work was based on the development of a two-balloon system that was used to perform soundings at sea on the ship “Princesse Alice” [25] in the early twentieth century. The balloon system used successfully in our measurement and described by Legain et al. [24] is a low cost device designed to be easily implemented on any free balloon sounding system to monitor the physico-chemical parameters and characteristics of the atmosphere. The balloon system was tested in 2011 (BLLAST for Boundary Layer Late Afternoon and Sunset Turbulence) and in Passy—France 2015 campaign [26]. The two-balloon (Figure 8a) system is referred to one carrier balloon and one slowing balloon in order to lift up the instrumentation (upward sounding at 5 m/s). Balloons are inflated using tares to ensure good repeatability of ascent and descent balloon speed. The carrier balloon is connected by a wire to the rawinsonde and its separation is triggered either at a specific atmospheric pressure previously fixed before the launch, or via a satellite communication system based on an Iridium element implemented on the AMULSE electronic board or at a specific timeout fixed in advance. When the carrier balloon is released, the instrumentation descends with the slowing balloon (downward sounding at 3.4 m/s was obtained through experimentation and proved to be a good compromise [24]). A meteorological radiosonde with a GPS probe fixed on the instrumentation and connected to a ground station allows the determination of the landing point which helps recovery of the instrumentation (Figure 8b). A flight simulation software is used to estimate the trajectories and the landing point of the probe. This estimation is based on the forecast vertical profile of horizontal wind with Météo-France operational Numerical Weather Prediction models Applications de la Recherche à l’Opérationnel à Méso-Echelle (AROME) [27,28] and Action de Recherche Petite Echelle Grande Echelle (ARPEGE) [29,30] at the launching site, the hypothesis on both the ascending/descending speed of the system and the release of the carrier balloon. A measured vertical profile of horizontal wind by the recent sounding can also be used. The trajectories of the balloon are updated in real time by the rawinsonde RS92 measured wind. Figure 9 shows the different stages of the AMULSE flight. In Figure 9a, the AMULSE is well fixed on the two-balloon system and ready to fly (Figure 9b). The two-balloon launch is shown on the Figure 9c. The pre-positioning (Figure 9d) determined previously by the flight simulation software allows us to recover the AMULSE (Figure 9e).

### 5.2. Performed Flights

The measurement campaign was held in France in the Champagne—Ardenne region, near Saint Hilaire le Grand (latitude 49°10′43.45″ N and longitude 4°28′3.72″ E) to be precise. During this measurement campaign organized in two periods (the first one from the 3rd to the 12th of September 2014 and the second one from the 22nd of September till the 3rd of October 2014) a total of 17 flights were carried out with six of them to test all the components while the rest where intended to measure the CO_2_ vertical profile up to 10 km altitude under Météo-France responsibility, flight direction and with the Meteorological Mobile Measurement Means facilities (4M, Météo France Institut National des Sciences de l’Univers INSU). Note that the pre-positioning system of Météo-France was 100% efficient so that we pre-localized the recovery site and had 100% recovery of the instrument.

**Figure 10:**
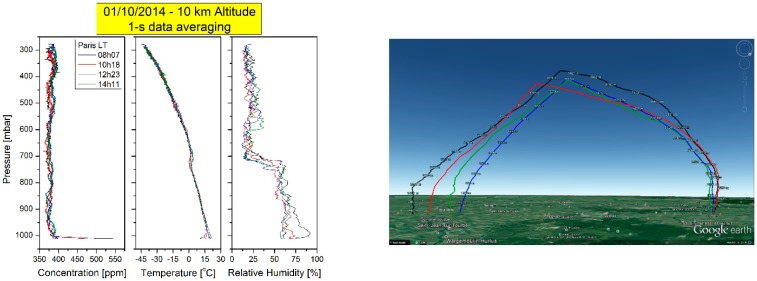
The four successive flights realized on 1 October 2014 launched from the same site from 08h07 till 14h11 Paris Local Time (approximately 2 h separating 2 successive flights). From left to right: Inverted pressure vs. the concentration to show the vertical profile of the CO_2_ up to 10 km altitude, the second panel is dedicated for pressure vs. temperature dependency, the third one is for relative humidity dependency. The fourth panel shows the launching site for all those flights and the recovery site according to the GPS position and plotted with the help of Google Earth.

The main objective of this campaign was to show the feasibility of measuring the CO_2_ profile during several periods of the day, in particular to study the boundary layer between early morning time and afternoon. A second objective was to slightly exceed the tropopause level in order to test the instrument in harsh environments. On 1 October 2014, we realized four successive flights up to 10 km altitude from the same launch site with the same instrument at 08h07, 10h18, 12h23 and 14h11 Paris Local Time. Figure 10 shows the results recorded in the upward sounding at a rate of 1 Hz (i.e., spatial resolution < 5 m), to the left, the inverted pressure scale vs. the concentration of the measured CO_2_, then the temperature according to the pressure and finally the relative humidity depending on the pressure. We also present the trajectories of these 4 flights with the help of Google Earth.

### 5.3. CO_2_ Evolution in the Atmospheric Boundary Layer

**Figure 11:**
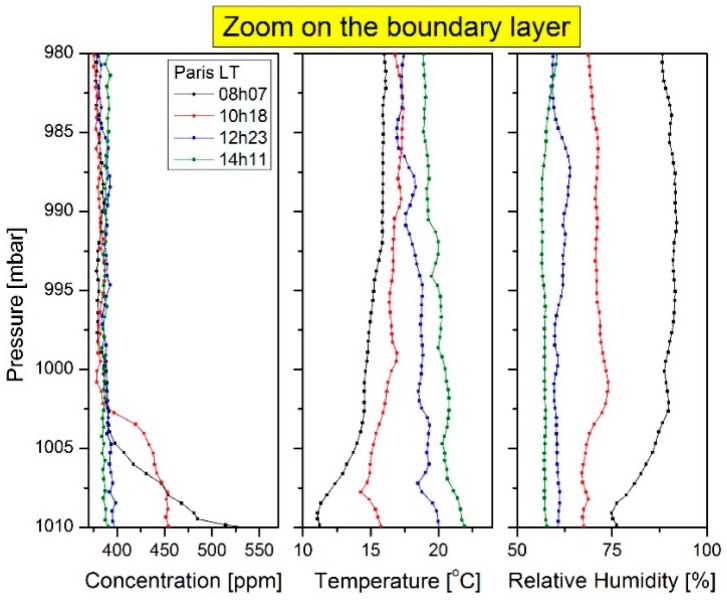
Zoom on the first 30 mbar of Figure 10 in order to study the boundary layer evolution during the same day. Results are in good agreement with the literature.

In order to better understand the boundary layer evolution, a zoom on the first 100 mbar was applied on the previous figure (Figure 11). Note that the blue line corresponds to the early morning measured data, the green line corresponds to the second flight, the red line is the third flight corresponding to the first afternoon flight. These observations are consistent with the literature. In the early morning, the concentrations of pollutants (more specifically of carbon dioxide) are found higher under the boundary layer compared to their amounts later in the day. This is due to the accumulation processes related to the nocturnal inversion [31]. Furthermore, the altitude of the boundary layer increases during the day along with dispersion and dilution processes that cause a decrease in pollutant concentrations.

## 6. Conclusions and Perspectives

A new compact lightweight spectrometer (1.8 kg) called AMULSE has been developed based on near-infrared laser technology and an open-path multipass cell. The spectrometer is dedicated to measurement of the vertical profiles of carbon dioxide in the atmosphere up to 10 km under meteorological and stratospheric balloons. We have demonstrated during the campaign high precision of 0.96 ppm for 1-s integration time (1σ), i.e., high temporal and spatial resolution (~1 Hz and 5 m). This instrument is autonomous, easy to use in a strap plug and play mode and is highly robust. Hence, the instrument was launched 17 times and was successfully recovered without any damage. The pre-localization prediction of Météo—France allowed prepositioning on the recovery site after each flight so a 100% instrument recovery rate was achieved.

The instrument optimization is underway and the newer version of the AMULSE will allow a simultaneous measurement of CH_4_ and CO_2_ up to 35 km altitude. This new dual-gas version of AMULSE will still complies with the light weights (<2.5 kg) required to be operated with weather balloons. It will furthermore feature real time onboard concentration retrieval. The preliminary results are promising with a precision of 0.3 ppm instead of 0.96 ppm in 1-s integration time for the CO_2_ channel based on 3 hours’ time series measurement and the Allan variance study (laboratory work). Several stratospheric flights are already programmed with this new version of AMULSE in Kiruna—Sweden (September 2016) and in Aire sur l’Adour—France (Fall 2016) with the CNES (Centre National d’Etudes Spatiales—Paris, France).
